# Gut microbiomes of wild and domesticated mammals and birds in Slovenia, Europe: 16S rRNA sequencing data

**DOI:** 10.1016/j.dib.2026.112564

**Published:** 2026-02-09

**Authors:** Zlender Tanja, Rupnik Maja

**Affiliations:** a Department for microbiological research, National laboratory of health, environment and food, 2000 Maribor, Slovenia; b Department of microbiology, Faculty of Medicine, University of Maribor, 2000 Maribor, Slovenia

**Keywords:** Gut microbiota, 16S rRNA gene, Metagenome, Animal feces

## Abstract

From a One Health perspective, the gut microbiota of animals acts as a major driver of microbial exchange between animals and the environment. Animals continuously release gut microbes into their surroundings, shaping environmental and human microbial communities and potentially dispersing pathogens. Characterizing gut microbiota across diverse animal hosts is therefore critical for understanding the patterns of microbial spread through ecosystems and their impact on animal, human and environmental health.

Here, we introduce a large, taxonomically diverse dataset of fecal microbiomes from 715 individual animals representing over 50 mammalian and avian species. We collected samples from both wild and domestic animals with an emphasis on capturing microbial diversity across a wide range of taxa and ecological contexts. The samples were subjected to 16S rRNA gene sequencing, targeting the V3–V4 hypervariable region. Bioinformatic analysis was performed using Usearch to generate zero-radius operational taxonomic units (ZOTUs).

This dataset was generated primarily for the development of microbial source tracking (MST) assays used for identifying the sources of fecal pollution in contaminated water. However, it provides a valuable resource for broader microbiome research. It enables comparative studies across host species, trophic guilds, and environmental contexts such as domestication.

Specifications Table

**Table:** 

Subject	Environmental genomics and metagenomics
Specific subject area	Gut microbiomes of mammals and birds
Type of data	Tables, figures, raw 16S rRNA sequences and analyzed ZOTU data
Data collection	A total of 715 mammalian and avian fecal samples were collected in Slovenia between December 2020 and September 2023. DNA was extracted and subjected to amplicon sequencing targeting the V3-V4 hypervariable regions of the 16S rRNA gene using next-generation sequencing (NGS) on the Illumina platform. Zero-radius operational taxonomic units (ZOTUs) were generated using Usearch and taxonomy assignment was performed with the RDP training set. The resulting ZOTU table was rarefied to 28,000 reads.
Data source location	Institution: National laboratory for health, environment and food (NLZOH) City: Maribor Country: Slovenia Latitude and longitude: Provided for each sample in Mendeley data repository (10.17632/b43trthrmg.2)
Data accessibility	Raw sequences Repository name: Sequence Read Archive (SRA) Data identification (accession) number: PRJNA1191222 Direct URL to data: https://dataview.ncbi.nlm.nih.gov/object/PRJNA1191222?reviewer=a1jbni6dtkjattgv7f3roh9ij9 Analyzed ZOTU data Repository name: Mendeley data Data identification number: 10.17632/b43trthrmg.2 Direct URL to data: https://data.mendeley.com/datasets/b43trthrmg/2
Related research article	Zlender T, Brezočnik L, Podgorelec V, Rupnik M. MicrobiomePrime: A primer pair selection tool for microbial source tracking validated on a comprehensive collection of animal gut and fecal waste microbiomes. Water Research 2026;289:124,990. https://doi.org/10.1016/j.watres.2025.124990.

## 1 Value of the Data

• This dataset provides a large collection of 16S sequencing data from fecal samples of 54 animal species, including some less-characterized hosts.
• The dataset provides information on animal fecal microbiomes of animals from a so far less covered geographical region.
• Important metadata for reuse include sample freshness, living conditions (wild/domestic), diet type, taxonomic ranks (species, genus, family, order, class), sampling location, key dates (sampling, freezing, sequencing), and sequencing platform/polymerase.
• Users can apply these metadata to filter samples (e.g. exclude DMVA samples or samples from captive animals) or account for potential biases (batch effects).
• The data enables comparative analyses of gut microbiota across diverse animal taxa and trophic guilds including alpha and beta diversity, differential abundance, functional prediction and taxonomic composition; exclusion of DMVA samples is recommended.
• The inclusion of animals from the same or closely related species under different living conditions allows for an investigation into how varying environments and domestication influence gut microbiota (e.g. wild boar versus domesticated pig and ibex versus domesticated goat).
• The dataset can be used for cross-host marker discovery, e.g. in library-independent MST.
• The dataset can serve as a reference library for library-dependent (community-based) MST approaches.

## 2 Background

Studying animal gut microbiota is crucial from a One Health perspective, as it is closely linked to environmental microbial ecosystems and the transmission of zoonotic diseases [1]. Moreover, animal gut microbiota represents an under-studied but important reservoir of microorganisms that can influence human microbiome composition through everyday human-animal interactions [2]. In this study, fecal samples from over 50 wild and domestic mammalian and avian species (totaling 715 samples) were collected primarily for the development of library-independent MST assays. These assays are designed to identify sources of fecal contamination in environmental, recreational, and drinking waters by detecting microbial nucleic acids unique to the feces of specific hosts, such as humans, livestock or wildlife. Such nucleic acids serve as biomarkers, which can be identified using a specially designed PCR assay [3,4]. While the MST application is discussed in a separate publication [5], this dataset also provides valuable insights into the gut microbiota of various animal species, some of which have not been well characterized. By understanding how diet, taxonomy, domestication, and habitat shape these communities, we can gain valuable insights for conservation strategies and agricultural practices, as well as public health management.

## 3 Data Description

This dataset consists of high-throughput sequencing data obtained from 515 mammalian and 200 avian fecal samples. In total, 51,345,923 sequences were generated from mammalian samples (mean = 99,700 ± 48,646 per sample) and 17,522,474 from avian samples (mean = 87,612 ± 60,465 per sample).

A summary of the mammalian fecal samples is shown in Table 1. Most samples originate from domesticated animals including cattle, pig, horse, sheep, goat, dog, cat, donkey and rabbit. However, there is also a significant number of wild mammalian fecal samples, especially from ungulates (different species of deer, ibex, chamois, wild boar) and rodents (nutria, beaver, mouse). Other wild animals sampled include hedgehogs, wild carnivorans (fox, otter, badger, bear) and bats. Some of the samples were collected from wild animals temporarily held in captivity for veterinary care, as well as from deer, which are being raised in controlled environments for meat production and tourism, as noted in Mendeley data.

**Table 1:** Overview of sampled animal groups from class *Mammalia*. The table includes group names, total sample counts, the number of samples after rarefaction, the number of DMVA samples, the number of sampling locations, and taxonomic classification at the genus, family, and order levels.

Group	Total samples	Rarefied samples	DMVA samples	No. Locations	Species	Genera	Families	Order
Cattle	79	79	2	17	*Bos taurus*	*Bos*	*Bovidae*	*Artiodactyla*
Ibex	15	15	11	2	*Capra ibex*	*Capra*	*Bovidae*	*Artiodactyla*
Sheep	14	14	0	5	*Ovis aries*	*Ovis*	*Bovidae*	*Artiodactyla*
Goat	13	13	3	6	*Capra aegagrus hircus*	*Capra*	*Bovidae*	*Artiodactyla*
Chamois	10	10	4	1	*Rupicapra rupicapra*	*Rupicapra*	*Bovidae*	*Artiodactyla*
Fallow deer	34	32	21	4	*Dama dama*	*Dama*	*Cervidae*	*Artiodactyla*
Roe deer	33	32	20	9	*Capreolus capreolus*	*Capreolus*	*Cervidae*	*Artiodactyla*
Red deer	17	17	14	3	*Cervus elaphus*	*Cervus*	*Cervidae*	*Artiodactyla*
Pig	45	45	4	16	*Sus scrofa domestica*	*Sus*	*Suidae*	*Artiodactyla*
Wild boar	17	17	2	4	*Sus scofa*	*Sus*	*Suidae*	*Artiodactyla*
Horse	36	36	4	12	*Equus ferus caballus*	*Equus*	*Equidae*	*Perissodactyla*
Donkey	2	2	0	2	*Equus africanus asinus*	*Equus*	*Equidae*	*Perissodactyla*
Beaver	15	15	5	1	*Castor fiber*	*Castor*	*Castoridae*	*Rodentia*
Nutria	23	23	9	4	*Myocastor coypus*	*Myocastor*	*Echimyidae*	*Rodentia*
Mouse	26	26	0	1	*Apodemus agrarius, Apodemus flavicollis, Apodemus sylvaticus, Apodemus* indet*.*	*Apodemus*	*Muridae*	*Rodentia*
Bat	9	7	6	9	*Rhinolophus hipposideros, Myotis myotis, Rhinolophus ferrumequinum,* *Eptesicus serotinus*	*Rhinolophus, Myotis, Eptesicus*	*Rhinolophidae, Vespertilionidae*	*Chiroptera*
Hedgehog	31	28	24	3	*Erinaceus concolor*	*Erinaceus*	*Erinaceidae*	*Erinaceomorpha*
Rabbit	4	4	0	3	*Oryctolagus cuniculus*	*Oryctolagus*	*Leporidae*	*Lagomorpha*
Dog	43	43	1	19	*Canis lupus familiaris*	*Canis*	*Canidae*	*Carnivora*
Cat	33	33	1	9	*Felis catus*	*Felis*	*Felidae*	*Carnivora*
Fox	4	4	0	2	*Vulpes vulpes*	*Vulpes*	*Canidae*	*Carnivora*
Otter	5	5	3	3	*Lutra lutra*	*Lutra*	*Mustelidae*	*Carnivora*
Badger	2	1	2	1	*Meles meles*	*Meles*	*Mustelidae*	*Carnivora*
Bear	3	3	1	2	*Ursus arctos*	*Ursus*	*Ursidae*	*Carnivora*
Unidentified	2	1	0	2	*Canidae* indet., *Carnivora* indet.	*Canidae* indet., *Carnivora* indet.	*Canidae, Carnivora* indet.	*Carnivora*

Samples with an undetermined host genus are labelled as “Unidentified”. The number of samples included in the analysis after rarefaction to 28,000 reads is indicated in the 'Rarefied Samples' column. Legend: DMVA – feces with decreased moisture content or visibly aged appearance, indet. – indeterminate, indicating that the taxon could not be identified beyond the listed taxonomic level.

The majority of avian fecal samples are from anatids (swan, duck, goose), columbids (pigeon, dove), gulls and domesticated birds (chicken, quail and turkey). The remaining samples include those from passerines and predatory birds including different species of owls, buzzard, kestrel and stork (Table 2). A proportion of birds sampled in our study (including all predatory birds) were held in captivity for veterinary care (Mendeley data).

**Table 2:** Overview of sampled animal groups from class *Aves*. The table includes group names, total sample counts, the number of samples after rarefaction, the number of DMVA samples, the number of sampling locations, and taxonomic classification at the genus, family, and order levels.

Group	Total samples	Rarefied samples	DMVA samples	No. Locations	Species	Genera	Families	Order
Chicken	35	35	0	15	*Gallus gallus domesticus*	*Gallus*	*Phasianidae*	*Galliformes*
Quail	4	4	0	1	*Coturnix coturnix*	*Coturnix*	*Phasianidae*	*Galliformes*
Turkey	2	2	0	1	*Meleagris gallopavo domesticus*	*Meleagris*	*Phasianidae*	*Galliformes*
Swan	43	38	5	4	*Cygnus olor*	*Cyngus*	*Anatidae*	*Anseriformes*
Duck	30	23	0	4	*Anas platyrhynchos, Anas platyrhynchos domesticus — Cairina moschata domestica, Anas indet.*	*Anas, Cairina*	*Anatidae*	*Anseriformes*
Goose	4	4	1	1	*Anser indet.*	*Anser*	*Anatidae*	*Anseriformes*
Gull	24	15	0	3	*Chroicocephalus ridibundus, Larus indet.*	*Chroicocephalus, Larus*	*Laridae*	*Charadriiformes*
Columbids	29	20	4	6	*Columbia livia domestica, Streptopelia decaocto*	*Columbia, Streptopelia*	*Columbidae*	*Columbiformes*
Passerines	7	1	0	1	*Parus major, Poecile palustris, Fringilla coelebs, Cyanistes caeruleus*	*Parus, Fringilla, Cyanistes*	*Paridae, Fringillidae*	*Passeriformes*
Predatory birds	13	5	1	1	*Athene noctua, Otus scops, Tyto alba, Strix uralensis, Buteo buteo, Falco tinnunculus, Strix aluco, Bubo bubo, Ciconia ciconia, Falco naumanni, Ciconia nigra*	*Athene, Otus, Tyto, Strix, Buteo, Falco, Bubo, Ciconia*	*Strigidae, Tytonidae, Accipitridae, Falconidae, Ciconiidae*	*Strigiformes, Accipitriformes, Falconiformes, Ciconiiformes*
Unidentified	9	9	0	1	*Aves* indet.	*Aves* indet.	*Aves* indet.	*Aves* indet.

Samples with an undetermined host genus are labelled as “Unidentified”. The number of samples included in the analysis after rarefaction to 28,000 reads is indicated in the 'Rarefied Samples' column. Legend: DMVA – feces with decreased moisture content or visibly aged appearance, indet. – indeterminate, indicating that the taxon could not be identified beyond the listed taxonomic level.

Sample sizes per group varied substantially, with some mammalian and avian groups represented by only a few samples. This variability should be considered when interpreting comparative analyses.

**Fig. 1:**
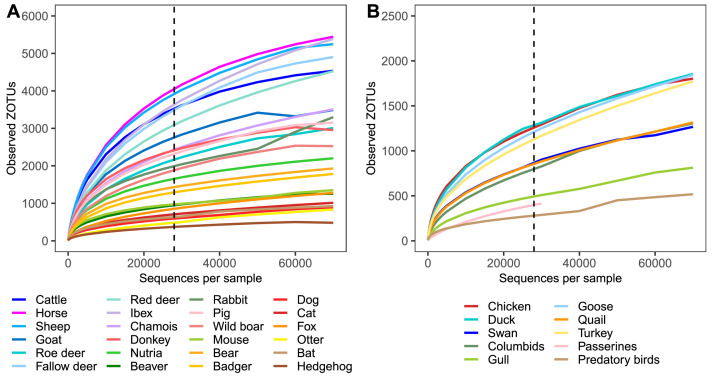
Mean rarefaction curves for animal groups. Mean rarefaction curves for animal groups from the orders (A) *Mammalia* and (B) *Aves*. Rarefaction depth selected for a general dataset overview is indicated by a black dashed line. Unidentified animal samples were excluded from this analysis.

The rarefaction curves for different animal groups approached a plateau at different points as illustrated for both mammals (Fig. 1A) and birds (Fig. 1B). Most mammalian samples displayed a general trend of capturing microbial richness at given sequencing depths. However, some fecal samples from animals in the order *Artiodactyla*, including cattle, horse, sheep, goat, ibex and different species of deer did not reach the plateau (Fig S1).

In comparison to mammalian samples, a significant proportion of avian samples had very low concentrations of DNA before sequencing, with some being under the detection limit (Mendeley data). This resulted in very low sequencing depths in some avian samples, with 30 out of 200 having fewer than 10,000 reads. Similarly to rarefaction curves of mammals, rarefaction curves for avian groups reached a plateau at different points (Fig. 1B). Only a small proportion of avian samples, primarily those with lower sequencing depth, failed to reach a plateau (Fig. S2).

For the following representation of this dataset, we rarefied the ZOTU table to 28,000 sequences per sample, excluding samples with fewer reads. This threshold was chosen to balance sample size and microbial diversity representation. The rarefaction resulted in the exclusion of 10 mammalian and 44 avian samples, among which 70 % of samples had DNA concentrations below 1 ng/µl and 48 % of samples had DNA concentrations under the detection limit.

**Fig. 2:**
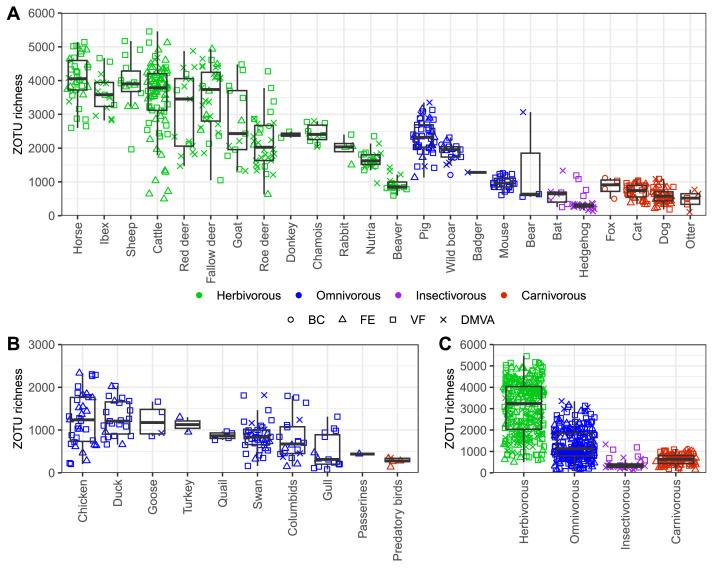
Alpha diversity analysis. ZOTU richness across (A) animal groups from the order *Mammalia*, (B) animal groups from the order *Aves* and (C) animals categorized by trophic guild. Unidentified animal samples were excluded from this analysis. Note that a part of samples (including all predatory bird samples) were obtained from wild animals in captivity, which may influence their microbial profiles. Legend: BC – bowel contents, FE – freshly excreted feces, VF – visibly fresh feces, DMVA – feces with decreased moisture content or visibly aged appearance.

**Fig. 3:**
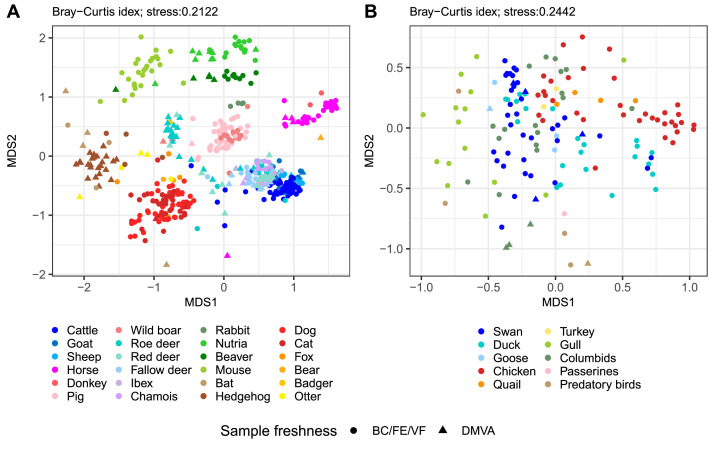
Beta diversity analysis. Visualization of beta diversity across samples from orders (A) *Mammalia* and (B) *Aves* using non-metric multidimensional scaling (NMDS) based on the Bray-Curtis distance matrix. Legend: BC – bowel contents, FE – freshly excreted feces, VF – visibly fresh feces, DMVA – feces with decreased moisture content or visibly aged appearance.

Alpha diversity was assessed using a measure of ZOTU richness, with results for individual animal groups and trophic guilds shown in Fig. 2. Notably, some samples with lower moisture content or a visibly aged appearance (e.g. from bear, bat, nutria, and hedgehog) stood out as outliers in comparison to freshly collected samples, a pattern also reflected in the beta diversity analysis (Fig. 3). With sample freshness specified in the metadata (Mendeley Data), any samples showing reduced moisture or aging characteristics (samples labeled as DMVA) can be easily excluded from the analysis, if needed.

**Fig. 4:**
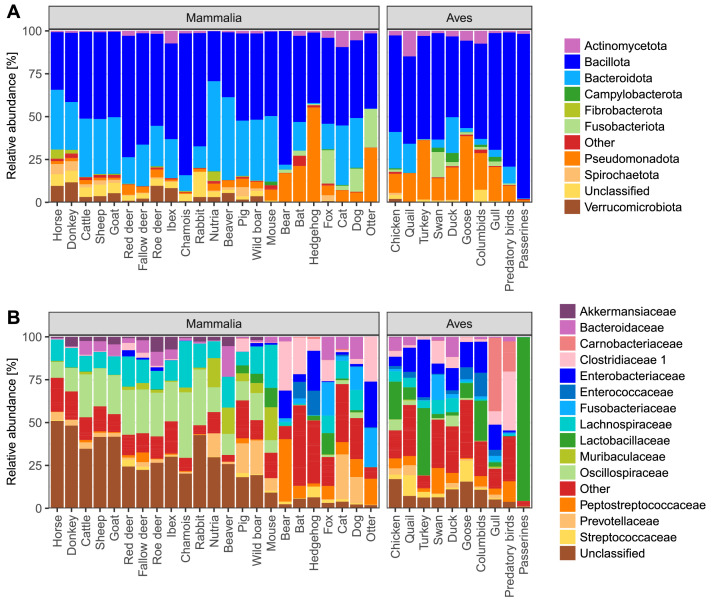
Microbial composition of gut microbiota in mammals and birds. The mean relative abundances of (A) bacterial phyla and (B) bacterial families in the gut microbiota of different groups of mammals and birds. DMVA samples and unidentified animal samples were excluded from this analysis.

In mammalian fecal samples, the predominant bacterial phyla were *Bacillota* and *Bacteroidota*. Although bacteria from phylum *Pseudomonadota* were present in all mammalian groups, they were especially highly abundant in hedgehogs, bats, bears and otters. Carnivorous mammals exhibited a visibly higher relative abundance of *Fusobacteria* compared to other trophic guilds (Fig. 4A).

On the family level, the most predominant bacterial taxa in herbivorous and omnivorous mammals were *Oscillospiraceae* and *Lachnospiraceae*. In rodents (nutria, beaver and mouse) there was a higher percentage of *Muribaculaceae* compared to other animal groups. The most predominant families in insectivorous animals were *Enterobacteriaceae, Enterococcaceae* and *Clostridiaceae*. In all carnivorous animals apart from otter, which feeds on fish, the predominant families were *Bacteroidaceae, Lachnospiraceae, Fusobacteriaceae* and *Peptostreptococcaceae*.

The most abundant bacterial phylum in bird samples was *Bacillota*. A notable proportion of *Pseudomonadota* and *Bacteroidota* was also observed in all bird groups except for passerines. On the family level, the most common bacterial taxa in avian feces were *Enterobacteriaceae, Clostridiaceae, Lactobactillaceae, Bacteroidaceae, Lachnospiraceae, Peptostreoticiccaceae and Streptococcaceae*. Bacteria from the family *Carnobacteriaceae* were especially highly abundant in gull and predatory birds.

**Fig. 5:**
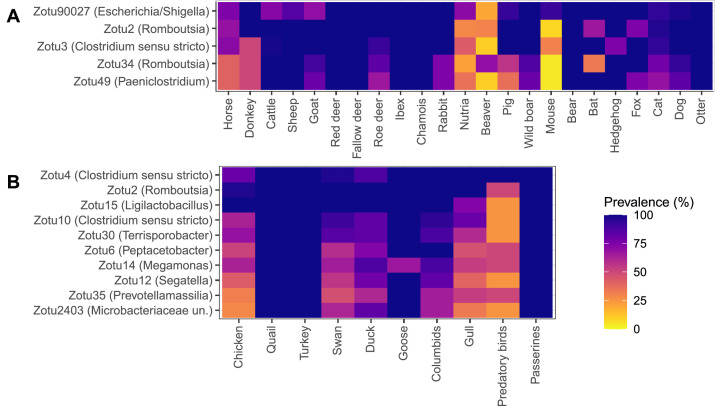
Core ZOTUs shared across mammalian and avian host groups. ZOTUs present in all animal groups within (A) order *Mammalia* and (B) order *Aves*, with a mean prevalence above 70 % across groups. Unidentified animal samples and DMVA samples were excluded from this analysis.

The core microbiome of animal orders *Mammalia* and *Aves* are presented in Fig. 5, showing ZOTUs that are present in all animal groups with a mean prevalence higher than 70 %. In mammals, the most prevalent ZOTUs were Zotu90027 (classified as *Escherichia/Shigella*) and Zotu2 (classified as *Romboutsia*). In birds, Zotu2 was also highly prevalent, alongside Zotu4 (classified as *Clostridium sensu stricto*).

### 3.1 Mendeley data repository structure

The Mendeley Data repository contains both raw and processed versions of the ZOTU table, ZOTU sequences in FASTA format, taxonomy table and sample metadata. The raw data is located in the "Raw" folder, while the processed data (filtered to remove non-bacterial sequences and rarefied) can be found in the "Filtered and rarefied" folder. All scripts used for data processing, analysis, and figure generation are provided in the “Code” folder. A complete list of all files and folders in the Mendeley data repository is provided in Table 3.

**Table 3:** Overview of folders and files in the Mendeley Data repository with brief descriptions.

**Folder**	**File name**	**Content**
Filtered and rarefied	metadata_rare.xlsx	Detailed metadata for samples retained after filtering and rarefaction
	taxonomy_rare.tsv	Taxonomic classification for each ZOTU retained after filtering and rarefaction
	zotus_rare.fa	Sequences of each ZOTU retained after filtering and rarefaction in a FASTA format
	zotutab_rare.tsv	Read counts of each ZOTU in each sample after filtering and rarefaction
Raw	metadata.xlsx	Detailed metadata for all samples included in the study
	taxonomy_raw.xlsx	Taxonomic classification for each ZOTU
	zotus_raw.xlsx	Sequences of each ZOTU in a FASTA format
	zotutab_raw.tsv	Read counts of each ZOTU in each sample
Code	usearch_script.sh	Script for running Usearch.
	filtering_and_rarefaction.R	R script for filtering and rarefying raw data generated by Usearch
	figure1_rarefaction_curves.R	R script for generating Figure 1
	figure2_boxplot.R	R script for generating Figure 2
	figure3_NMDS.R	R script for generating Figure 3
	figure4_microbiota_composition.R	R script for generating Figure 4
	figure5_core_microbiome.R	R script for generating Figure 5
	supplementary_figures.R	R script for generating Figures S1 and S2

The raw data consists of a ZOTU table (zotutab_raw.tsv), ZOTU sequences in FASTA format (zotus_raw.fa), a corresponding taxonomy file (taxonomy_raw.tsv), and sample metadata (metadata_raw.xlsx).

The processed dataset has been filtered to remove non-bacterial and chloroplast sequences and rarefied to 28,000 reads. Samples with fewer than 28,000 reads were removed. This version also includes a rarefied ZOTU table (zotutab_rare.tsv), sequences in FASTA format (zotus_rare.fa), a corresponding taxonomy file (taxonomy_rare.tsv), and sample metadata (metadata_rare.xlsx).

The metadata files include detailed information on each individual sample including animal taxonomy, breed (if applicable), trophic guild, age and sex of the animal, sample freshness, sampling location, sampling dates, as well as the dates of sample freezing, DNA isolation, and sequencing. It also provides the information on animal living conditions (wild/domestic), with wild animals further classified into one of the three categories: free-ranging, temporarily captive for veterinary care, and bred and raised in captivity. Furthermore, the metadata provides the details about the polymerase and sequencing platform used.

## 4 **Experimental Design, Materialsand Methods**

### 4.1 Sampling

Fecal samples wild and domestic mammals and birds were collected across Slovenia between December 2020 and September 2023. In total, we obtained 515 mammalian and 200 avian fecal samples. The samples were collected using sterile stool containers with a plastic spoon and immediately stored in ice-filled coolers. Where possible, only the interior of the fecal deposit was sampled to minimize contamination from external sources such as soil, litter and urine. The samples were transported to the laboratory within 24 h. When immediate transport was not possible, they were stored at −20 °C and later transported to the laboratory on ice. The samples were stored at −80 °C until further processing.

### 4.2 DNA isolation and amplicon sequencing

The DNA was isolated from 0.25 g of each sample using the QIAamp Fast Stool Mini Kit (Qiagen, Hilden, Germany) with mechanical disruption (MagNA Lyser; 7000 rpm for 70 s). The concentrations of DNA were measured using the Quant-iT PicoGreen dsDNA Kit (Thermo Fisher Scientific). The DNA was stored at −80 °C until further processing.

The V3-V4 hypervariable region of the 16S rRNA was amplified with the primers Bakt_341F (5′-CCTACGGGNGGCWGCAG-3′) and Bakt_805R (5′-GACTACHVGGGTATCTAATCC-3′). Library preparation followed the Illumina 16S Metagenomic Sequencing Library Preparation protocol (Illumina, CA, USA) and was performed using either KAPA HiFi HotStart ReadyMix (Kapa Biosystems, MA, USA) or Q5 High-Fidelity DNA Polymerase (New England Biolabs, USA). After each PCR during library preparation, amplicons were run on a gel alongside a negative control, and no contamination was detected.

Sequencing was carried out on Illumina platforms (MiSeq or NextSeq 2000) using 600-cycle reagent kits. Batch effects may occur due to the use of two sequencing platforms (MiSeq, NextSeq) and two polymerases (KAPA HiFi HotStart ReadyMix, Kapa Biosystems; Q5 High-Fidelity DNA Polymerase, New England Biolabs). Relevant information is provided for each sample in the metadata_raw.xlsx file (Mendeley Data), allowing these effects to be accounted for during downstream analysis. A 10 % PhiX control was included in each run for quality control.

Raw sequencing data is available in the NCBI SRA under accession number PRJNA1191222.

### 4.3 Raw sequence analysis

Raw sequences were processed using Usearch version 11.0.667 [6,7]. Paired-end reads were merged with the maximum of 15 mismatches in the alignment. Primer sequences were removed and sequences exceeding one expected error were discarded. Unique sequences underwent denoising with the UNOISE algorithm, generating zero-radius operational taxonomic units (ZOTUs). Sequences shorter than 400 bp were excluded, and taxonomy was assigned using the RDP training set (v.19) [8].

### 4.4 Filtering and normalization

Further filtering was performed using R version 4.2.3 [9]. Taxa with confidence scores below 0.8 were categorized as unclassified. Non-bacterial ZOTUs, and sequences classified as chloroplasts at the class level were excluded from the dataset. The ZOTU table was rarefied to 28,000 reads per sample using the Vegan package v.2.6.4 [10] with seed set to 28. Samples with <28,000 reads were excluded.

### 4.5 Animal grouping for microbial composition analysis

To explore patterns in microbial composition, we grouped animals into broad trophic guilds based on their predominant dietary tendencies: herbivorous, omnivorous, carnivorous, and insectivorous. While some species exhibit dietary flexibility, these categories reflect general feeding ecology trends.

Additionally, we grouped the samples into broader taxonomic or ecological categories to simplify data presentation. Details on group classifications, sample counts, and associated taxonomic levels for each animal group after rarefaction are provided in Table 1, while information for individual samples can be found in the metadata_raw.xlsx file (Mendeley Data).

### 4.6 Visualizations and analysis

We analyzed the microbial community data using R v4.2.3 [9]. Rarefaction curves and core microbiome calculations were performed on raw data from samples with ≥28,000 reads. Chloroplasts and non-bacterial sequences were excluded for the core microbiome analysis. All other analyses and visualizations used filtered, rarefied data. The visualizations were created using ggplot2 v3.4.2 [11]. Alpha diversity was calculated as the number of unique ZOTUs per sample after rarefaction. Beta diversity was then assessed by generating a non-metric multidimensional scaling (NMDS) plot based on the Bray-Curtis distance matrix, using the vegan package v2.6.4 [10].

The core microbiome was determined at the ZOTU level for each animal order (*Mammalia* and *Aves*) separately, using filtered data after excluding non-bacterial sequences. ZOTUs that were present in all animal groups and had a mean prevalence greater than 70 % were considered part of the core microbiome. To calculate mean prevalence, we first determined the prevalence of each ZOTU within each animal group. The mean prevalence was then calculated across all groups.

Unidentified animal samples were excluded from all analyses. DMVA samples were further excluded from microbial composition and core microbiome analyses, as they tended to show different alpha and beta diversity patterns compared with other freshness categories.

## Limitations

- Uneven sample sizes across species/animal groups.
- Variability in sample freshness, which appears to strongly influence microbial diversity and composition (DMVA samples, defined as feces with decreased moisture content or a visibly aged appearance, can be easily excluded from the analysis).
- Low sequencing depth in some samples (especially avian).
- Temporal span of sampling could introduce biases related to seasonal variation in microbial communities.
- The dataset was generated using two different polymerases and Illumina sequencing platforms, which may introduce variability in sequencing results.

## **Ethics Statement**

The authors confirm that they have read and follow the ethical requirements for publication in Data in Brief. The current work does not involve human subjects, animal experiments, or data collected from social media platforms.

According to national guidelines, ethical approval was not required for the collection of animal fecal samples. Most samples were collected from the ground, if possible, immediately after defecation. Bowl contents were obtained post-mortem from some wild animals after being hunted down by members of the Slovenian Hunters Association. No animals were harmed or subjected to disruptions in their daily activities for the purposes of this study.

## CRediT Authorstatement

**Tanja Zlender**: Investigation, Formal analysis, Writing – Original Draft, Visualization. **Maja Rupnik**: Conceptualization, Resources, Writing – Review & Editing, Supervision, Funding acquisition.

## Data Availability

Mendeley DataGut microbiomes of mammals and birds from Slovenia: 16S rRNA sequencing data (Original data).

## Appendix Supplementary materials

Image, application 1
