# Supplementary material for: Gut microbiomes of wild and domesticated mammals and birds in Slovenia, Europe: 16S rRNA sequencing data
Source: Data Brief. 2026 Feb 9;65:112564. doi: 10.1016/j.dib.2026.112564 (PMC12925480; doi:10.1016/j.dib.2026.112564)
Supplement: Supplementary file 1 [file mmc1.pdf]

Supplementary information

Gut microbiomes of wild and domesticated mammals and birds in Slovenia, Europe: 16S rRNA sequencing data

Zlender Tanja[1,2*], Rupnik Maja[1,2]

[1]Department for Microbiological Research, National Laboratory of Health, Environment and Food, 2000 Maribor, Slovenia

[2]Department of Microbiology, Faculty of Medicine, University of Maribor, 2000 Maribor, Slovenia

*Correspondence to tanja.zlender@nlzoh.si

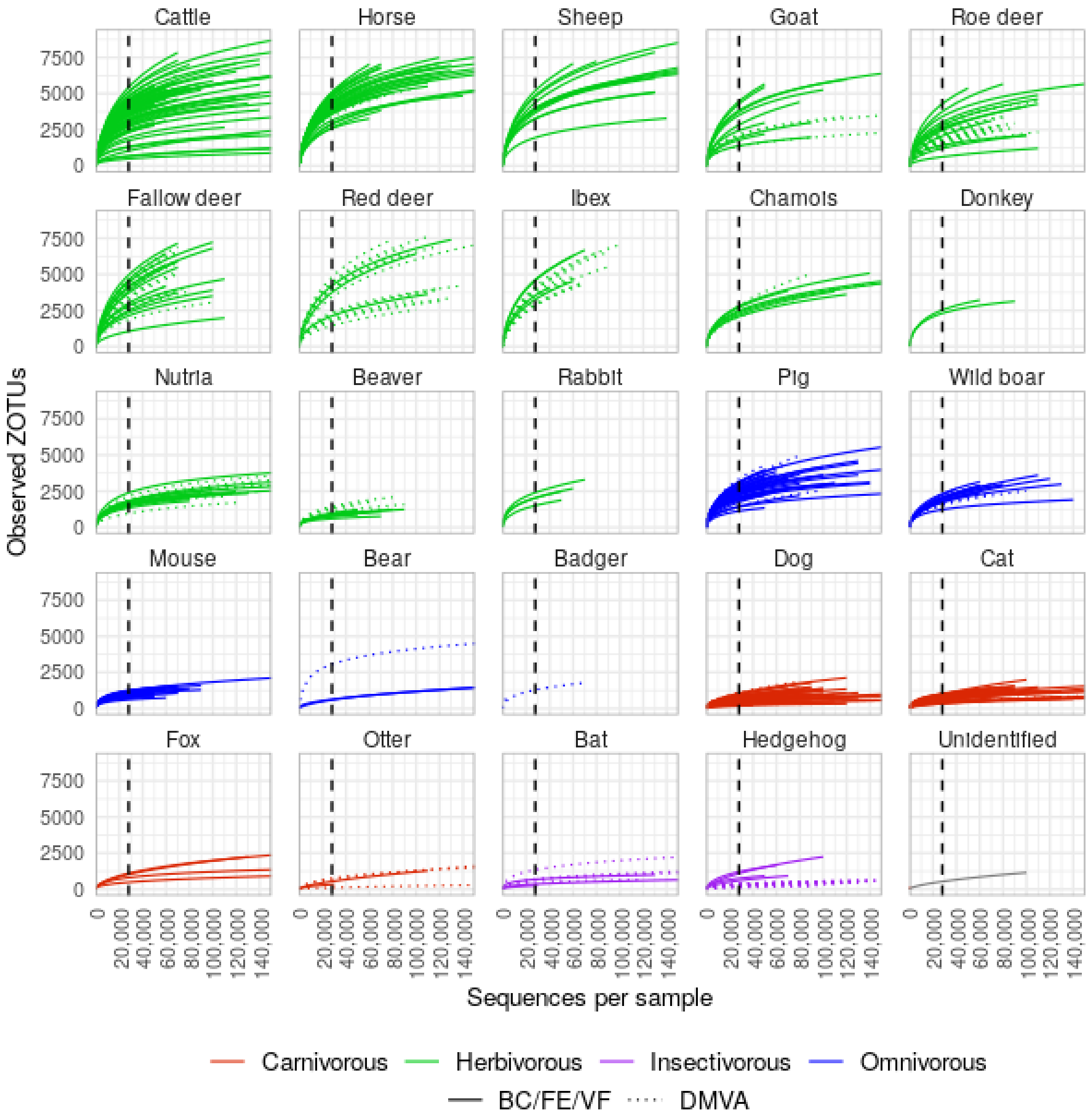

Figure S1: Rarefaction curves for mammalian samples, categorized by animal group. Each curve represents a separate sample, with different animal groups displayed in distinct panels. Rarefaction depth selected for a general dataset overview is indicated by a black dashed line. Legend: **BC** – bowel contents, **FE** – freshly excreted feces, **VF** – visibly fresh feces, **DMVA** – feces with decreased moisture content or visibly aged appearance.

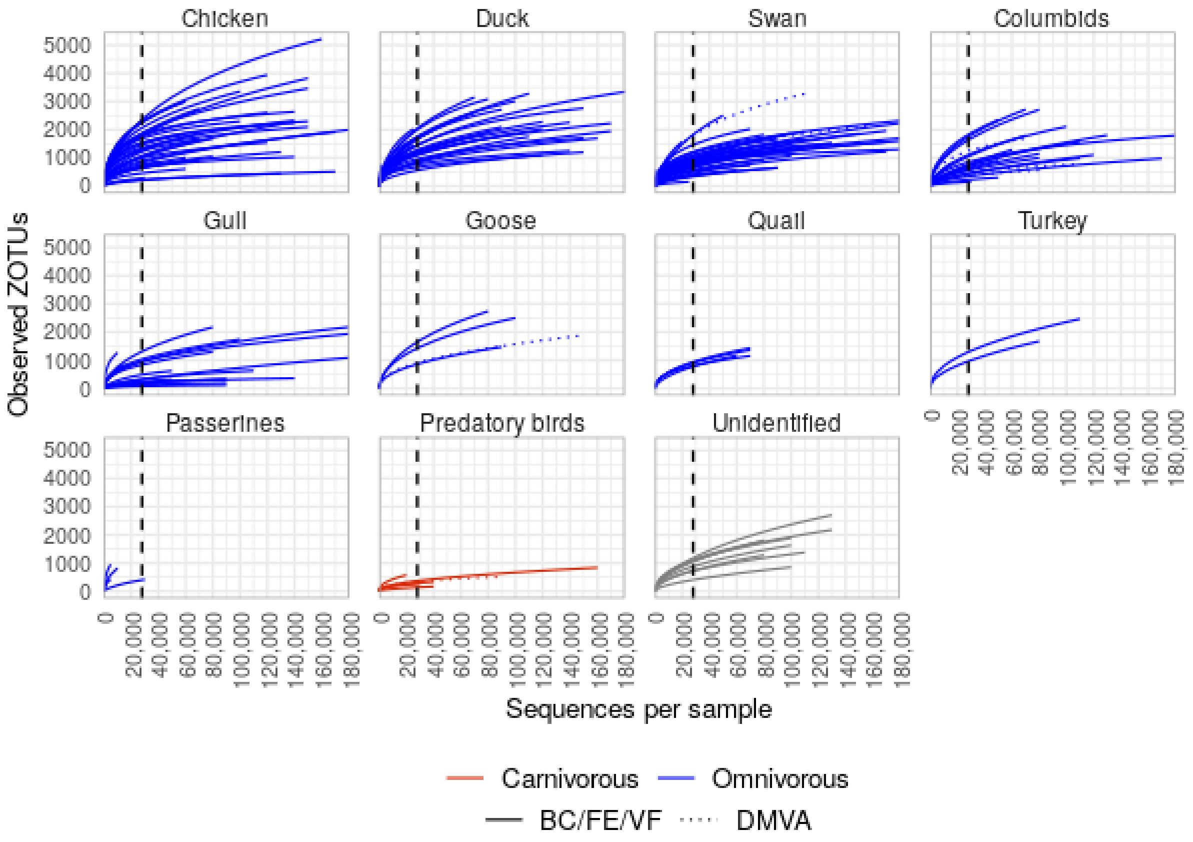

Figure S2: Rarefaction curves for avian samples, categorized by animal group. Each curve represents a separate sample, with different animal groups displayed in distinct panels. Rarefaction depth selected for a general dataset overview is indicated by a black dashed line. Legend: **BC** – bowel contents, **FE** – freshly excreted feces, **VF** – visibly fresh feces, **DMVA** – feces with decreased moisture content or visibly aged appearance.
